# Cutaneous mucormycosis caused by *Saksenaea vasiformis* in a patient with systemic lupus erythematosus

**DOI:** 10.1002/ccr3.1698

**Published:** 2018-07-13

**Authors:** Liyanage Shamithra Madhumali Sigera, Kavinga Kalhari Kobawaka Gamage, Melani Naamal Jayawardena, Wijewickrama Punchihewage Harshula Abeydeera, Matharage Ariyalatha Malkanthi, Primali Irosha Jayasekera, Charini Geethika Upulkanthi Arukattu Patabendige, Arumabaduge Harshini Niranjali Fernando

**Affiliations:** ^1^ Department of Mycology Medical Research Institute Colombo Sri Lanka; ^2^ National Hospital Colombo Sri Lanka

**Keywords:** cutaneous mucormycosis, identification difficulties, *Saksenaea vasiformis*, systemic lupus erythematosus

## Abstract

Primary cutaneous mucormycosis due to *Saksenaea vasiformis* is a rare clinical manifestation and the actual number of the disease condition is underestimated due to lack of sporulation in the absence of molecular diagnosis. Combination therapy of antifungal and repetitive debridement is mandatory in curing the patients.

## 1 INTRODUCTION

Mucormycosis is an opportunistic fungal infection caused by fungi of the order Mucorales in the class Mucormycetes.^1^ The commonest manifestation of mucormycosis is rhinocerebral form (39%).^2^, ^3^ However, mucormycosis has a variety of other clinical presentations including pulmonary, gastrointestinal, disseminated, and cutaneous.^3^ Approximately 19% of the mucormycosis are cutaneous form and the associated mortality of cutaneous mucormycosis is around 16%.^2^, ^4^ Cutaneous mucormycosis follows the introduction of the fungal spores through any interruption of the protective skin due to trauma, surgery, burn, laceration, insect bite, abrasion, or intravenous injection.^2^

*Rhizopus* spp.*, Lichtheimia corymbifera* spp.*, Rhizomucor* spp.*, Mucor* spp.*, Apophysomyces* spp.*,* and *Saksenaea* spp. have been identified as causative agents of primary cutaneous mucormycosis, where *Rhizopus* ranks the top of the list.^5^ *S. vasiformis* is isolated relatively infrequently as an agent for cutaneous mucormycosis and approximately 40 cases of human *Saksenaea* mucormycete infections have been reported up to now.^6^, ^7^, ^8^

However, the actual number of cases must have been underestimated due to identification problems associated with this fungal species.^6^, ^7^, ^8^

Here, we present a patient with cutaneous mucormycosis caused by *S. vasiformis* which was successfully treated with intravenous antifungal drugs and repeated debridement in the setting of SLE.

## 2 CASE REPORT

A 29‐year old female diagnosed with SLE for 4 years complicated with grade II lupus nephritis presented with status epilepticus. She denied a history of fever on admission, but was treated with cyclophosphamide 1 month prior for an episode of cerebral lupus. She had noticed a papule over the left deltoid region which progressed to an ulcer over 1 week. Fever was noted following several days of hospital admission and the ulcer site became painful. She had worked in paddy fields several months prior to the admission when she was in good health. However, she could not recall any precipitating injury at the affected site during working. She is a mother of two and both pregnancies were uncomplicated. She denied history of alcohol abuse or smoking.

**Figure 1:**
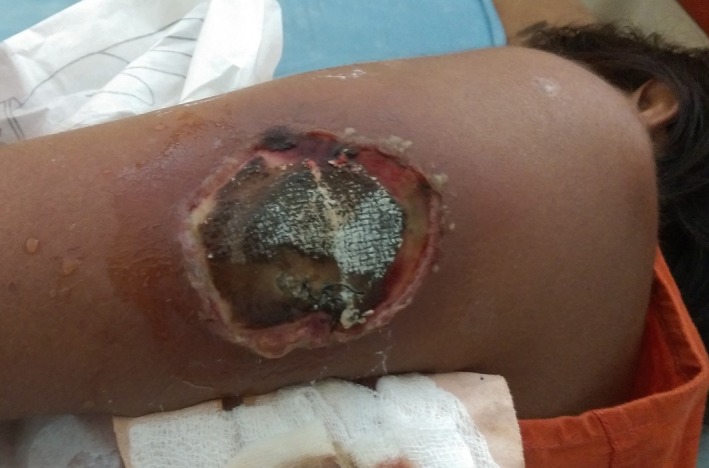
The ulcer with the necrotic center and surrounding erythema

On examination she was emaciated and had a GCS score of 15/15 following recovery of status epilepticus. There was no obvious lymphadenopathy. At presentation, the size of the ulcer was about a 3 cm lesion and it gradually developed in to an ulcer with a necrotic center with surrounding erythema. A tentative diagnosis of pyoderma gangrenosum was made with the appearance of the ulcer (Figure 1). It gradually advanced into the underlying muscle over 3 weeks of onset despite the antibiotic treatment. Examination of the cardiovascular, respiratory systems, and the abdomen was normal.

Her full blood count, blood picture, and other supportive investigations showed evidence of microangiopathic hemolytic anaemia, which was suggestive of thrombotic thrombocytopenic purpura which resolved following plasmapheresis. Her ESR was persistently normal. Renal functions were stable during hospital stay, so were the liver profile. Chest radiography revealed evidence of bilateral mild pleural effusions and echocardiography revealed a thin rim of pericardial effusion and good cardiac function. MRI, MRA brain showed evidence of Posterior Reversible Encephalopathy Syndrome. Repeat imaging showed resolved changes.

**Figure 2:**
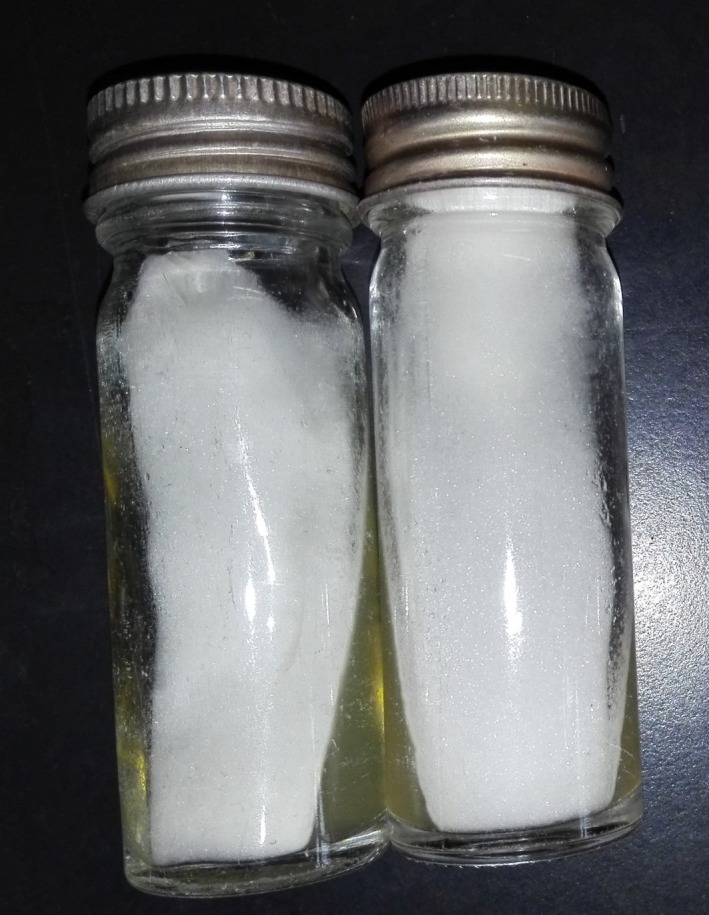
White aerial mold in culture bottles

A punch biopsy of the skin was done from the lesion and sent for fungal studies and histopathological studies. The direct microscopy examination revealed wide and irregular ribbon‐like nonseptate hyphae with right‐angle branching suggestive for Mucormycete fungi. Culture was done on Sabouraud dextrose agar with chloramphenicol (at 26°C and 37°C) yielded a white aerial mold, which covered the entire surface of the agar and came up to the lid of the culture bottles after 4 days of incubation (Figure 2).

The lactophenol cotton blue mount of the growth revealed broad, nonseptate hyaline sterile hyphae. The slide culture test has been attempted with the hope of sporulation, however it was not successful. They only resulted in broad, nonseptate hyaline sterile hyphae without spores. Then the isolate was subcultured on to potato dextrose agar (PDA) and Rose Bengal (RB) agar for induction of sporulation. However, they yielded only sterile mycelia.

**Figure 3:**
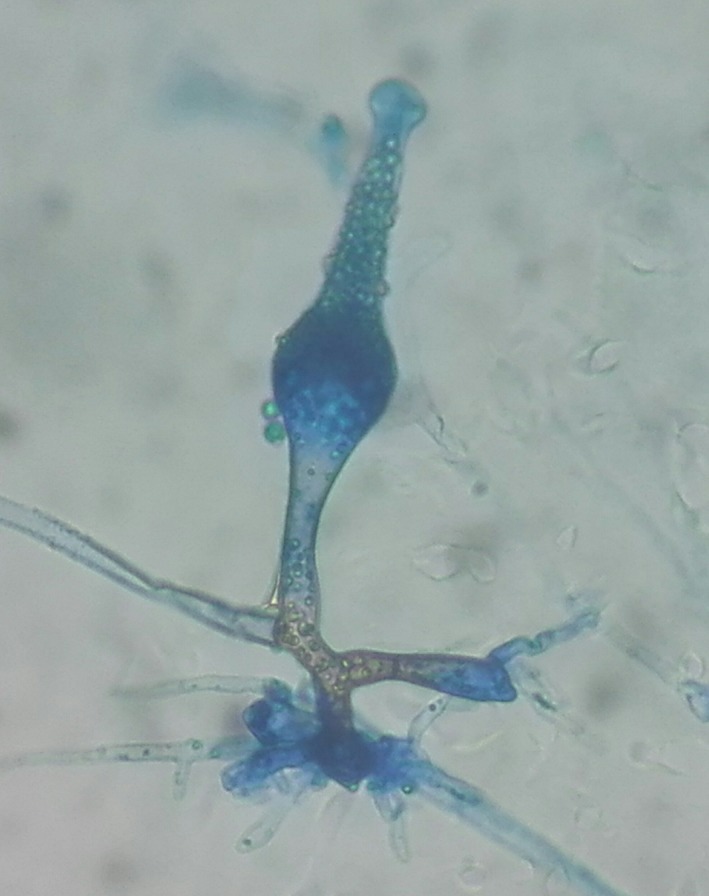
Lactophenol cotton blue mount showing characteristic flask‐shaped sporangium in short sporangiophore with rhizoids

The isolate was inoculated on nutritionally deficient medium, tap water agar and incubated for 14 days at 37°C. It provided a hazy view of flask shaped sporangium with rhizoids in lactophenol cotton blue mount. Then floating agar method was used and it yielded characteristic flask‐shaped sporangium in short sporangeophore with rhizoids after 10 days of incubation (Figure 3).The sporangia had a long neck and the apex of the neck closed with a mucilaginous plug. The sporangiospores were cylindrical, with rounded ends. Those morphological features were suggestive for *S. vasiformis* and the isolate was identified as *S. vasiformis*.

The histopathology of the punch biopsy of the skin also reveled broad aseptate hyphae suggestive of Mucormycetes group of fungi.

Based on the histopathological evidence of broad aseptate hyphae, suggestive of Mucormycete fungi, the patient was started on IV amphotericin B deoxycholate. Repeated surgical debridement was done and samples were sent for fungal studies. However, local application of antifungals was not included in the management. Her second tissue biopsy, which was taken during debridement after 5 days of IV amphotericin B also had similar direct microscopy findings and yielded *S. vasiformis*. However third tissue sample which was obtained after 10 days after IV amphotericine B deoxycholate became negative for fungal studies. Following the confirmation of sterile cultures from the subcutaneous biopsies, superficial skin grafting was done which was completely accepted from the wound site. She was treated with intravenous conventional amphotericin B for 28 days and she was asymptomatic when she was discharged from the ward.

## 3 DISCUSSION

*Saksenaea vasiformis* is a fungus of the family Saksenaeaceae included in order Mucorales of class Zygomycetes.^1^ Even though *S. vasiformis* has been considered the only member of genus *Saksenaea*, four new species of the genus, *S. vasiformis, S. erythrospora, S. oblongispora*, and *S. loutrophoriformis* have been identified recently, based on physiological, morphological, and molecular characteristics.^6^, ^9^, ^17^, ^18^

Similar to other members of the order Mucorales, *S. vasiformis* produces hyaline, broad fungal filaments.^15^ However, the most characteristic feature of the organism is it's sporulation.^10^ They produce single, unbranched, sporangiophores with dichotomously branched, darkly pigmented rhizoids.^15^ These sporangiophores bear single, terminal multispored, typical flask‐shaped sporangia.^15^ The sporangiospores are smooth walled and ellipsoidal to cylindrical.^15^

*Saksenaea vasiformis* was first isolated from soil in India by Saksena in 1953.^7^, ^11^ Thereafter, this filamentous fungus has been isolated from soil samples, driftwood, and grains, from different parts of the world including India, United States, Central America, and Israel.^9^, ^10^, ^12^

It has been reported as etiological agent for both human and veterinary infections.^9^, ^10^ There are reports of fatal cutaneous lesions caused by these fungi among dogs, cow, and dolphins.^10^ The first human infection due to this fungi was described by Ajello et al^13^ Since then patients have been reported from tropical and subtropical countries including Central and South America, United States, New Zealand, French Guiana, Australia, Thailand, Tunisia, India, and the Middle East.^5^, ^7^

Approximately 40 cases of human *Saksenaea* mucormycetes infections have been reported up to now, however the actual number of cases must have been underestimated due to identification problems associated with this fungi.^6^, ^7^, ^8^

In Sri Lanka, P.Perera has described the first case of *S. vasiformis* infection in a 1‐year‐and‐6‐month‐old female child, who presented with a nodule on the right side of the bridge of the nose, orbital cellulitis and fever. The diagnosis was confirmed by microscopic examination and culture of the material obtained from the retro‐orbital space. The patient was treated with amphotericine B with complete resolution of the infection.^16^

Unlike other mucormycosis, *S. vasiformis* infection is largely limited to the skin and subcutaneous tissue and most of the reported cases are cutaneous infections.^7^, ^9^, ^14^ The cutaneous infections follow the traumatic disruption of the integrity of the skin and usually seen after burns, tattoos, insect bites, scorpion bites, motor traffic accidents, lacerations, and abrasions.^4^, ^5^, ^9^, ^10^ It has also been reported following needle stick injuries, intramuscular injections, vascular catheterization, and surgery in healthcare sector.^3^, ^9^ Our patient had worked in paddy fields several months prior to the admission when she was in good health. Even though she could not recall any precipitating injury at the affected site while working, the traumatic disruption of the skin may have been followed by the infection.

*Saksenaea vasiformis* skin and subcutaneous infections commonly present as necrotizing fasciitis or rapidly spreading cellulitis.^9^ Although *Saksenaea* species are not dependent on the underling predisposing factors, immunocompromised individuals, similar to our patient, are appeared to be more frequently affected by necrotizing fasciitis or rapidly spreading cellulitis.^9^ Our patient had been treated with cyclophosphamide, a potent immunosuppressive agent, for an episode of cerebral lupus which may have contributed to the progression of her condition. These are frequently localized lesions and have favorable outcome with surgical debridement and effective antifungal therapy.^9^ However, they can be complicated with bacterial superinfections or progress rapidly despite antifungal treatment.^9^ Similar to other members of the order Mucorales, *S. vasiformis* infection is evident by angioinvasion leading to thrombosis and tissue necrosis.^9^ This facilitates the progression of infection into deep tissue.^6^

*Saksenaea vasiformis* also reported as chronic primary cutaneous or subcutaneous infections.^9^ They appear as erythematous papules or nodules which gradually increase in size over months.^9^ These lesions could be either painless or painful and may be associated with low‐grade fever and regional lymphadenopathy.^9^ This type is frequently reported in otherwise healthy males.^9^

Other than cutaneous and subcutaneous infections, *S. vasiformis* has been responsible for other forms of disease presentations.^9^ *S. vasiformis* has been reported infrequently as a cause of rhino‐orbito‐cerebral, pulmonary, or disseminated infections.^1^, ^12^, ^14^ Pierce et al^8^ has described a case of osteomyelitis in a young man following a crush injury. *S. vasiformis* has been recovered from endocardium, lungs, skin lesions, and thyroid during postmortem in a fatal disseminated case.^12^ Hematogenous dissemination to distance organs after inhalation of spores could be the most probable mode of infection in these rare disseminated cases.^9^, ^10^, ^14^ The prognosis of these manifestations is poor than cutaneous form and most of the patients with rhino‐orbito‐cerebral infections (83%) and disseminated infections (75%) are dying.^9^

Diagnosis of cutaneous mucormycosis is difficult unless clinicians have high degree of clinical suspicion.^2^ Deep tissue biopsy is the most appropriate specimen.^2^ It should be evaluated for fungal elements by direct smear and fungal culture (to identify the causative organism) and histologically. A ribbon‐like, folded, widely aseptate hypae with the evidence of vascular invasion, hemorrhage, infarction, and thrombosis will be observed in histopathology.^2^

Direct microscopy with 10% KOH reveals wide and irregular ribbon‐like nonseptate hyphae and right‐angled branching suggestive for Mucormycetes fungi.^15^

This fungus easily grows in routine fungal culture media^4^ and produce expanding colony within few days.^9^, ^15^ However, they fail to produce spores as other Mucorales members.^4^ In microscopic examination of culture with lactophenol cotton blue, it appears as sterile, broad, aseptate, wide hyaline hyphae.^9^, ^15^ Poor sporulation in common mycological media affects negatively in accurate identification of this fungus.^10^

Sporulation should be induced by nutritionally deficient medium such as Czapek's agar, Borelli's lactrimel agar, or saline agar.^4^ An efficient sporulation has also been observed using special culture techniques such as distilled water method or floating agar block method.^4^ Induction of sporulation has succeeded with the tap water agar and exposure to sunlight.^4^ Microscopic examination of sporulated culture reveals vase‐shaped sporangia and dark rhizoids, characteristic of the *S. vasiformis* complex.^9^

In the absence of rapid sporulation method, molecular techniques are used for the identification of *S. vasiformis*.^5^ These molecular methods are based on internal transcribed spacer (ITS) sequencing and PCR amplification.^3^ Different molecular targets are used to identify fungi directly from frozen or paraffin‐embedded tissues.^5^

Effective antifungal therapy, repeated aggressive surgical debridement, and correction of underline predisposing condition, are mandatory for the survival of the patient.^3^ IV amphotericin B is the most widely appreciated antifungal drug either alone or combined with posaconazole.^9^ There are reports of antifungal susceptibility testing showing high minimum inhibitory concentrations (MICs) for voriconazole, amphotericin B, echinocandins, and low MICs for itraconazole, posaconazole, and terbinafine.^3^ Alvarez et al^7^ found that both amphotericin B (1‐8) and voriconazole (2‐8) had high MIC values in *S. vasiformis*. They found that low MIC values for itraconazole (0.06‐1), posaconazole (0.06‐0.25), and terbinafine (0.03‐1). The low MIC value for posaconazole suggests the clinical utility of this antifungal drug against *S. vasiformis* infections.^9^

## 4 CONCLUSION

In summary, we report a case of cutaneous *Saksenaea vasiform* is infection which was successfully managed with IV amphotericine B and surgical debridement. When nonsporulating Mucormycetes is isolated from a specimen, *Saksenaea* spp or *Apophysomyces* spp should be suspected. These fungi should be cultured in specific culture media to induce sporulation.

## CONFLICT OF INTEREST

None declared.

## AUTHOR CONTRIBUTION

LSMS, MAM and MNJ: carried out laboratory experiments for the identification of *Saksenaea* from the cultured biopsy specimen. KKKG, AHNF, PIJ, CGUAP, and WPHA: involved in clinical management of the patient and provided patient information for the manuscript. LSMS and KKKG: wrote the manuscript. PIJ and CGUAP: supervised on laboratory procedures and manuscript writing.

## CONSENT FOR PUBLICATION

Written informed consent was obtained from the patient for the publication of this case report and any accompanying images. A copy of the written consent is available for review upon requested.

## AVAILABILITY OF DATA AND MATERIAL

The data that support the findings of this study are available from the corresponding author upon reasonable request.
